# Comparison of Abbreviated MRI and Full Diagnostic Protocol MRI for Surgical Planning in Patients with Newly Diagnosed Breast Cancer

**DOI:** 10.3390/diagnostics15212749

**Published:** 2025-10-30

**Authors:** Seo Young Park, Hyejin Cheon, Won Hwa Kim, So Mi Lee, Ji Young Park, Hye Jung Kim

**Affiliations:** 1 Department of Radiology, School of Medicine, Kyungpook National University, Kyungpook National University Chilgok Hospital, 807 Hoguk-ro, Buk-gu, Daegu 41404, Republic of Korea; uniun0926@knu.ac.kr (S.Y.P.);; 2 Department of Pathology, School of Medicine, Kyungpook National University, Kyungpook National University Chilgok Hospital, Daegu 41404, Republic of Korea; jyparkmd@knu.ac.kr

**Keywords:** breast cancer, breast MRI, diagnostic imaging, tumor burden, pathology

## Abstract

**Objective**: This study aimed to compare the concordance of abbreviated MRI (AB-MRI) and full diagnostic protocol MRI (FDP-MRI) with pathology in assessing tumor extent for surgical planning in patients with newly diagnosed breast cancer. Additionally, we evaluated the performance of AB-MRI and FDP-MRI in detecting additional malignant lesions in the ipsilateral breast. **Materials and Methods**: A total of 319 patients with 330 index breast cancers were enrolled in the study. Two radiologists independently assessed tumor extent on AB-MRI and FDP-MRI and compared their measurements with the pathological tumor extent. For both MRI protocols, concordance rates and agreement of tumor extent with pathology were analyzed according to histopathologic and molecular subtypes using the chi-square test and ICC. Additional malignant lesions detection rates in the ipsilateral breast were compared between the two MRI protocols. **Results**: The mean total pathologic tumor extent, including the in situ component and adjacent malignant lesions, was 2.2 cm (standard deviation [SD]: 1.3 cm; range: 0.7–8.5 cm). The concordance rate of tumor extent with pathology between the two MRI protocols (AB- and FDP-MRI) showed no significant difference (reader 1, *p* = 0.68; reader 2, *p* = 0.74). The agreement of tumor extent with pathology was not significantly different between the two MRI protocols (AB-MRI and FDP-MRI: K = 0.70, 0.75, *p* = 0.17 in reader 1; K = 0.65, 0.71, *p* = 0.15 in reader 2). The detection rate of additional malignant lesions showed no significant difference between AB-MRI and FDP-MRI (*p* = 0.71 in reader 1, *p* = 0.89 in reader 2). **Conclusions**: AB-MRI is comparable to FDP-MRI for assessing tumor extent and detecting additional malignant lesions.

## 1. Introduction

Magnetic resonance imaging (MRI) of the breast has the highest sensitivity in detecting breast cancer compared to mammography or ultrasound [1]. The usage of breast MRI has expanded to preoperative cancer staging, monitoring neoadjuvant chemotherapy, and cancer screening [2,3,4]. Moreover, it is a sensitive imaging modality for detecting additional lesions that cannot be detected by mammography in breast cancer [5].

Despite the superior diagnostic capability of MRI, its usage is somewhat limited by high costs and longer image acquisition and interpretation times, especially for full diagnostic protocol MRI (FDP-MRI). Actually, undergoing FDP-MRI lasts about 30–40 min; therefore, staying in the prone position during the scan can be uncomfortable, which might lead to motion artifacts. To overcome these limitations, Kuhl et al. proposed an abbreviated MRI protocol (AB-MRI) for screening [6]. During AB-MRI, the contrast enhancement study is performed only at two specific times (before and after contrast media administration during the first phase) [7], substantially shortening image acquisition and interpretation times.

To date, most previous studies have verified that the diagnostic accuracy of AB-MRI in cancer screening is equivalent to that of FDP-MRI [8,9]. Additionally, if breast cancer is incidentally detected through AB-MRI, some questions may arise about whether FDP-MRI should be performed, especially for assessing tumor extent for surgical planning. Given these circumstances, we hypothesize that AB-MRI may have a potential role in assessing tumor extent in the preoperative setting. Therefore, the primary objective of this study is to compare the concordance rates and agreement of AB-MRI and FDP-MRI with pathology for assessing tumor extent. Additionally, the study aims to compare the performance of AB-MRI and FDP-MRI in detecting additional malignant lesions in the ipsilateral breast.

## 2. Material and Methods

### 2.1. Study Population

**Figure 1:**
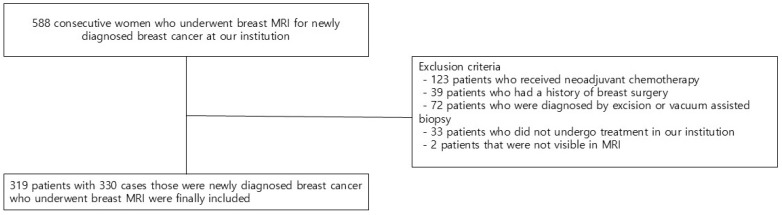
Flow chart of the study population.

We reviewed the electronic medical records of 588 consecutive patients who underwent breast MRI for newly diagnosed breast cancer. Of the 588 patients, those who initially received neoadjuvant chemotherapy (*n* = 123), those who had previous breast surgery (*n* = 39), those who were diagnosed by excision or vacuum-assisted biopsy (*n* = 72), those who did not undergo treatment in our institution (*n* = 33), and those with tumors not visible on MRI (*n* = 2) were excluded. Finally, 319 patients with 330 cases (11 cases of bilateral cancer) were included in the study (Figure 1).

### 2.2. Imaging Techniques

MRI (Discovery MR750; GE Healthcare, Waukesha, WI, USA, 3T) was performed using a dedicated eight-channel surface breast coil in the prone position. Gadobutrol contrast agent (0.1 mL/kg; Gadovist, Bayer Schering Pharma, Berlin, Germany) was intravenously administered at a rate of 1 mL/s. The Supplementary Table S1 shows more details of the acquisition protocol. Subtraction images and 3D maximum intensity projection (MIP) images were also generated. To simulate the AB-MRI protocol for this study, we selected a subset of sequences (composed of pre-contrast T1, first post-contrast T1, MIP, along with T2-weighted images) from the full protocol. T2-weighted imaging was included in the simulated AB-MRI protocol to improve specificity by distinguishing T2-hyperintensity suggesting benignity from abnormal enhancement, thus allowing additional lesion characterization.

The approximate scan times for FDP-MRI and AB-MRI were around 30 and less than 10 min, respectively.

### 2.3. Image Analysis

Two breast imaging radiologists (H.J.C. and W.H.K., with 2 and 12 years of experience, respectively) independently assessed the breast MRIs with an interval of at least one-month wash-out between AB-MRI and FDP-MRI. The order of interpretation of each MRI protocol was randomized. The readers only knew that they were patients with breast cancer.

**Figure 2:**
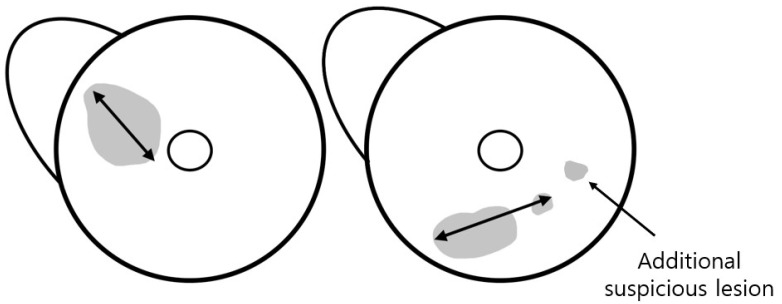
Assessment of tumor extent using MRI. The total tumor extent was measured as the maximum extent of the index lesion, including suspicious lesions within 15 mm of the index cancer. An additional suspicious lesion was defined as a lesion located >15 mm away from the index tumor.

The total tumor extent was determined by the maximum extent of the index lesion. For mass, the maximum dimension was measured along the largest enhancing margin, whereas for non-mass enhancement (NME), the extent was defined as the largest linear dimension including whole enhancement areas. In cases with combined mass and NME, the total extent was measured as the longest dimension encompassing both components. When satellite suspicious lesions without continuity with the index lesion were located within 15 mm of the index cancer, the total extent including these lesions was measured (Figure 2). The two readers also documented anatomical location of the tumor (left or right, clock position, and distance from the nipple). They also assessed breast density, background parenchymal enhancement (BPE) on the first post-contrast T1-weighted images, and reading time for tumor detection and measurement of the total tumor extent in each reading mode.

The readers evaluated the presence of additional lesions in the ipsilateral breast and categorized images based on the Breast Imaging Reporting and Data System (BI-RADS) for MRI [10]. We defined an additional suspicious lesion as a lesion located more than 15 mm away from the index tumor, based on the study by van Loevezijin et al. [11], as such lesions were considered unlikely to be included in the wide local excision of the index tumor with a safe surgical margin. Furthermore, a lesion was established as any finding assigned a category of ≥4 by each reader on each MRI protocol. Those lesions were matched between MRI and pathology by two other breast radiologists (S.Y.P. and H.J.K., with 2 and 23 years of experience, respectively) who had not participated in the reading study. Findings included in the biopsy or surgical specimen were classified as benign or malignant based on the pathological results.

### 2.4. Histopathological Analysis

The pathological tumor extent was defined as the largest dimension of the lesions, including any in situ component and adjacent cancer within 15 mm of the index tumor, according to the pathological reports. In addition to pathological tumor extent, histopathologic subtypes and immunohistochemical status, including estrogen receptor (ER), progesterone receptor (PR), human epidermal growth factor receptor 2 (HER-2), and Ki-67, were evaluated. The Supplementary Material shows the evaluation criteria for ER, PR, HER-2 expression, and Ki-67. Using immunohistochemistry, the tumors were classified into the following four subtypes according to hormone receptor (HR [ER or PR]) and HER-2 expression status: Luminal A (hormone receptor-positive, *HER-2*-negative, low Ki-67), Luminal B (hormone receptor-positive, *HER-2*-negative, high Ki-67), *HER-2* (*HER-2*-positive regardless of the hormone receptor status), and the triple-negative (TN) subtype (both hormone receptor and *HER-2*-negative) [12].

### 2.5. Statistical Analysis

The difference in tumor extent between the two MRI protocols was calculated by subtracting the tumor extent measured in AB-MRI from that measured in FDP-MRI. The discrepancy in tumor extent between MRI and pathology was assessed by subtracting the tumor extent measured on pathology from that measured on each MRI protocol. *p*-values were analyzed using the one-sample, one-sided Wilcoxon rank-sum test with the alternative hypothesis that the mean difference exceeds 10. The mean and 95% confidence interval were estimated.

Concordance between the tumor extents obtained from each MRI and the pathological tumor extents was defined as a difference within 10 mm [5], and the *p*-values were calculated using a chi-square test.

The agreement of tumor extent between the two MRI protocols and between each MRI and pathology was assessed using intraclass correlation coefficient (ICC) analysis. The ICC-defined agreements between the measurements of each MRI protocol and pathology as poor (0.00–0.20), fair (0.21–0.40), moderate (0.41–0.60), good (0.61–0.80), and excellent (0.81–1.00).

Spearman’s rank correlation coefficients were used to assess the correlation between tumor extents measured by each MRI protocol and pathology according to histopathological and molecular subtypes.

Cancer detection rate (CDR), positive predictive value (PPV), and false detection rate (FDR) were compared between the two reading modes for diagnosing ipsilateral additional malignant lesions. The McNemar test was also used to compare the CDR, PPV, and FDR.

The CDR was calculated as the percentage of correctly identified additional malignant lesions relative to the total number of pathologically confirmed additional cancers. The PPV was determined as the percentage of correctly identified additional malignant lesions to the total number of BI-RADS ≥ 4. The FDR was derived as the percentage of false positives to the total number of BI-RADS ≥ 4.

All statistical analyses were performed using MedCalc software (version 20.114, MedCalc). A *p*-value < 0.05 was considered statistically significant.

## 3. Results

### 3.1. Patients’ and Tumor Characteristics

Table 1 presents patients’ and tumor characteristics of the pathologically confirmed cancer lesions. The ages of the 319 included patients ranged from 27 to 78 years (mean: 51.5 ± 4.5). Out of these, 182 (58%) patients exhibited symptoms associated with malignancy, such as palpable mass or nipple discharge, whereas 137 (42%) patients had no symptoms. Among 330 breast cancer cases, 219 (68%) were invasive ductal carcinoma (IDC), 36 (11%) were ductal carcinoma in situ (DCIS, *n* = 5 in Van Nuys grade 1, *n* = 31 in Van Nuys grade 2, 3), and 45 (14%) were invasive lobular carcinoma. Other cancer types (*n* = 30) included mucinous carcinoma (*n* = 8), metaplastic carcinoma (*n* = 7), microinvasive papillary carcinoma (*n* = 6), malignant phyllodes tumor (*n* = 6), tubular carcinoma (*n* = 2), and secretory cancer (*n* = 1). Regarding molecular subtypes, 145 (44%) cases were Luminal type A, 124 (37%) cases were Luminal B, 25 (8%) cases were HER-2-positive cancer, and 36 (11%) cases were TN cancer. The lesion morphology on MRI was as follows: mass in 242 cases (73.3%), NME in 58 cases (17.6%), and combined mass with NME in 30 cases (9.1%). Of all breast cancer cases, 89 (27%) cases underwent total mastectomy, while 241 (73%) cases underwent breast-conserving surgery. The mean total pathologic tumor extent, including the in situ component and adjacent malignant lesions, was 2.2 cm (standard deviation [SD]: 1.3 cm; range: 0.7–8.5 cm). Among the 330 lesions, 34 lesions (10.4%) measured less than 1 cm in total pathologic extent.

### 3.2. Comparison of the Total Tumor Extent of AB-MRI and FDP-MRI with Pathology

In the one-sample Wilcoxon rank-sum test, the tumor extent differences between each MRI protocol and pathology did not significantly differ (all *Ps* = 1.00) when the mean difference was set at 10. To investigate the relationship between patient and tumor characteristics and MRI-based tumor extent measurement, logistic regression analysis was performed. Univariable analysis showed significant associations of menopausal status, breast density, and BPE with MRI tumor extent measurement (*p* < 0.05), but none remained significant in the multivariable analysis (*p* > 0.05).

The concordance rates between each MRI and pathology are shown in Table 2. The concordance rate of tumor extent between AB-MRI and pathology showed no significant difference compared to that between FDP-MRI and pathology for all tumor subtypes. In the evaluation according to histopathologic subtypes, IDC revealed the highest rate of correct estimation (AB-MRI and FDP-MRI: 88.1% and 89.0%, reader 1; each 88.6%, reader 2), whereas DCIS demonstrated the lowest rate of correct estimation (58.3% and 55.6%, reader 1; 55.6% and 58.3%, reader 2). In DCIS groups, overestimation was more frequent compared to other histopathologic subgroups (27.8% and 33.3%, reader 1; each 33.3%, reader 2). On the other hand, ILC exhibited the highest incidence of underestimation (17.8% and 13.3%, reader 1; 15.6% and 13.3%, reader 2). Regarding molecular subtypes, TN cancer exhibited the highest correct estimation rate (each 97.2%, reader 1; 100% and 97.2%, reader 2), while HER-2 cancer groups showed the lowest correct estimation rate (48.0% and 52.0%, reader 1 and 2, respectively). HER-2 cancer was characterized by the highest incidence of both overestimation and underestimation when compared with other molecular subtypes.

The ICC for the agreement of tumor extent between the two MRI protocols was good for overall cases (K = 0.79, reader 1; 0.74, reader 2; *p* = 0.12). The agreement between each MRI protocol and pathologic examination was also good for the total cases (AB-MRI and FDP-MRI: K = 0.70 and 0.75, *p* = 0.17 in reader 1; K = 0.65 and 0.71, *p* = 0.15 in reader 2). The agreement of tumor extent between AB-MRI and pathology was not significantly different from that between FDP-MRI and pathology (Table 3) for all tumor subtypes. For histopathologic subtypes, other tumor types showed the highest agreement (AB-MRI and FDP-MRI: 0.88 and 0.92, reader 1; 0.81 and 0.85, reader 2), whereas ILC showed the lowest agreement (0.52 and 0.60, reader 1; 0.49 and 0.62, reader 2). For molecular subtypes, TN showed the highest agreement (0.85 and 0.87, reader 1; 0.88 and 0.90, reader 2).

Spearman’s rank correlation coefficients showed a significant correlation between each MRI and pathology for tumor extent: 0.75 and 0.73 for readers 1 and 2, respectively, with AB-MRI, and 0.72 and 0.75 for readers 1 and 2, respectively, with FDP-MRI. The “other” tumor type showed the highest correlation coefficient (AB-MRI and FDP-MRI: 0.73 and 0.80, reader 1; 0.75 and 0.77, reader 2), while ILC showed the lowest correlation coefficient (0.49 and 0.60, reader 1; 0.47 and 0.58, reader 2) in histopathologic subtypes. In molecular subtypes, TN showed the highest correlation coefficient (0.79 and 0.81, reader 1; 0.81 and 0.84, reader 2). The *p*-values for comparing the correlation coefficients of the two MRI protocols with pathology in all subgroups were >0.41. Hence, the correlation of AB-MRI with pathology was not statistically different from that of FDP-MRI with pathology across all subgroups (Table 4).

The mean interpretation time was 38.17 s and 69.40 s (SD = 15.41 s, 21.50 s, *p* < 0.01) in AB-MRI and FDP-MRI, respectively.

### 3.3. Diagnostic Performance for Additional Ipsilateral Malignant Lesions

Table 5 describes the diagnostic performances of AB-MRI and FDP-MRI for additional ipsilateral malignant lesions. In patients with suspicious lesions in the ipsilateral breast, 15 pathologically proven additional malignant lesions were identified. Of the 15 additional malignant lesions, 11 (73.3%) presented as mass, 3 (20.0%) as NME, and 1 (6.7%) as combined morphology. Pathologically, these included 10 IDC, 2 DCIS, and 3 ILC (Supplementary Table S2). Two of the fifteen malignant lesions were not detected on AB-MRI by both readers, although those were found on FDP-MRI. They appeared as an oval, circumscribed mass of 5 mm (IDC) and a linear non-mass lesion of 17 mm (DCIS) with initial slow and delayed plateau enhancement.

Although there were no significant differences in CDR, PPV, and FDR between AB-MRI and FDP-MRI (*p* = 0.71 and 0.89 for readers 1 and 2 in CDR; *p* = 0.9 and 0.77 for readers 1 and 2 in PPV; and *p* = 0.52 and 0.12 for readers 1 and 2 in FDR), all three parameters (CDR, PPV, and FDR) tended to be lower with AB-MRI compared to FDP-MRI.

## 4. Discussion

This retrospective study evaluated the concordance and agreement of AB-MRI and FDP-MRI protocols with pathology in assessing tumor extent for surgical planning in patients with newly diagnosed breast cancer. Unlike previous studies, this study compared AB-MRI and FDP-MRI in assessing tumor extent across both histopathologic and molecular subtypes. Regarding the evaluation of tumor extent, AB-MRI did not provide statistically different results compared to FDP-MRI.

Multiple studies [13,14,15] demonstrated comparable diagnostic performance across different AB-MRI protocols in cancer detection. In particular, Lee-Felker et al. [16] compared AB-MRI with FDP-MRI for detecting multifocal and multicentric lesions in evaluating newly diagnosed breast cancer, revealing that AB-MRI with mammography or ultrasound achieved an acceptable agreement with FDP-MRI. Building upon this, our study measured the total tumor extent on MRI, including any suspicious nodules located within 15 mm of index cancer, which is considered during the initial surgical planning for the primary tumor. Moreover, this study evaluated differences in tumor extent assessment according to histopathologic and molecular subtypes.

The concordance rate with pathology was highest in IDC and TN and lowest in DCIS and HER-2. The highest concordance rate of TN cancer is that it is strongly associated with high tumor grade and unifocality, and its tendency to form a mass rather than spread into the tissue [17]. DCIS showed the lowest concordance rate (55.6–58.3%) and highest overestimation rate (27.8–33.3%) in histopathologic subtypes, due to prone positioning during breast MRI causing elongation of tumor extent [18]. This positional effect may impact the accuracy of tumor extent assessment, particularly for less dense tumors, such as DCIS. These overestimations of disease extent could prompt more aggressive surgery than necessary, such as an unnecessary mastectomy [19].

Interestingly, while the correct estimation rate was low (55.6–58.3%) in DCIS, the ICC was notably high (0.77–0.81). We believe this indicates that while MRI measurements may not precisely match the pathological tumor extent, they may still maintain a consistent pattern in relative tumor extent comparisons. Furthermore, the ICC may be overestimated due to greater variability in the small sample size of DCIS (*n* = 36). According to Koo T.K. et al. [20], sample size can significantly influence the reliability of the ICC, with small sample sizes compromising its accuracy. Therefore, a larger study is needed to validate this result.

HER-2 cancer demonstrated the lowest concordance rate among molecular subtypes. Although the mechanism by which HER-2 overexpression reduces the accuracy of breast MRI is unclear, the high degree of tumor angiogenesis and the permeability of the neovascular structures can lead to overestimation of tumor extent [21], and the presence of microcalcifications may lead to underestimation of tumor extent on MRI [22].

While the overestimation rate was higher than the underestimation rate in most subtypes, the underestimation rate was highest (13.3–17.8%), and the agreement was the lowest (0.49–0.60) in ILC for all histopathologic subtypes. These results align with those observed by Pereskycha et al. [23]. Apparently, this type of cancer grows in a single-file pattern, infiltrates the stroma without cell adherence, and rarely induces a desmoplastic reaction. Therefore, these distinct growth patterns may lead to underestimation of tumor extent assessed by MRI [24]. However, the ILC’s infiltrative growth pattern can lead to an underestimation of lesion extent on imaging, which may result in incomplete excision and tumor-positive margins requiring reoperation [25].

Regarding the diagnostic performance of identifying additional malignant lesions in the ipsilateral breast, despite tending to be lower with AB-MRI compared to FDP-MRI, CDR, PPV, and FDR were not statistically different between the two MRI protocols in both readers. Similarly, E.S. Kim et al. [26] revealed that the overall performance of AB-MRI was comparable to FDP-MRI in differentiating between benign and malignant lesions. In our study, although there was no statistically significant difference between AB-MRI and FDP-MRI, AB-MRI tended to show lower diagnostic performance, suggesting the need for a larger study to confirm these findings.

One notable challenge identified in our study was that two additional malignant lesions—one small mass (5 mm, IDC) and one linear NME (17 mm, DCIS)—were not detected on AB-MRI but were visible on FDP-MRI. These findings underscore a potential limitation of abbreviated protocols, especially in detecting small or slow-enhancing lesions. A potential way to address this limitation in clinical practice is to adopt a tailored imaging approach. After initial AB-MRI detects the index lesion, selective omission of FDP-MRI can be considered in specific scenarios—such as IDC or TN breast cancers, which have shown the highest concordance rate or ICC between MRI and pathology.

This study has several limitations. First, it was a single-center study that involved a limited patient sample in each histopathological and molecular tumor subtype. Second, our study measured the maximum tumor extent including the MIP images, identifying solitary lesions and multifocal cancer. However, the plane used to measure the tumor diameter in MRI differed from the plane used for specimen slicing in pathology, which might have affected the tumor extent measurements in both MRI and pathology [27]. Third, simulated AB-MRI in our study included T2-weighted imaging; however, this resulted in a longer imaging acquisition time compared to other AB-MRI protocols without T2-weighted imaging. Moreover, the inclusion of T2-weighted imaging in our simulated AB-MRI protocol may have resulted in the reduction of false positives. Therefore, direct comparisons with minimal AB-MRI studies should be made carefully because of differences in imaging protocols. Fourth, in our study, mammography and ultrasound were not included in tumor extent measurement; however, based on Lee-Felker et al. [15], we speculate that incorporating additional imaging modalities may further improve the diagnostic accuracy of AB-MRI.

In conclusion, our study suggests that preoperative AB-MRI could offer a comparable concordance rate and agreement in measuring pathologic total tumor extent, as well as equivalent performance in evaluating additional malignant lesions in the ipsilateral breast compared to FDP-MRI. Therefore, AB-MRI can be a feasible alternative for surgical planning, particularly for patients with mild claustrophobia or physical limitations, offering shorter imaging and reading times, thus improving efficiency and cost-effectiveness.

## Figures and Tables

**Table 1:** Patients’ and tumor characteristics.

Patient Characteristics	N = 319
Patient age	51.5 years ± 4.5
Menopausal status	
premenopause	136 (42%)
postmenopause	183 (58%)
Breast density	
almost entirely fat	15 (6%)
scattered fibroglandular tissue	34 (10%)
heterogeneous fibroglandular tissue	250 (78%)
extreme fibroglandular tissue	20 (6%)
BPE	
minimal	161 (50%)
mild	85 (26%)
moderate	60 (19%)
marked	13 (5%)
Mode of detection	
screening	137 (42%)
diagnostic	182 (58%)
Tumor Characteristics	N = 330
Subtype	
IDC	219 (68%)
DCIS	36 (11%)
ILC	45 (14%)
others	30 (9%)
Tumor extent	
≤2 cm	214 (65%)
2–5 cm	103 (31%)
≥5 cm	13 (4%)
ER status	
positive	262 (79%)
negative	68 (21%)
PR status	
positive	246 (75%)
negative	84 (25%)
HER-2 status	
positive	25 (7%)
negative	305 (93%)
Ki-67	
Low (<14%)	179 (54%)
High (≥14%)	151 (46%)
Molecular subtype	
Luminal A	145 (44%)
Luminal B	124 (37%)
HER-2	25 (8%)
TN	36 (11%)
MRI morphology	
mass	242 (73.3%)
NME	58 (17.6%)
mass with NME	30 (9.1%)
Surgery	
mastectomy	89 (27%)
BCS	241 (73%)

Note: data are the number of patients with percentages in parentheses. Others refer to the following: mucinous (8), metaplastic (7), microinvasive papillary carcinoma (6), malignant phyllodes tumor (6), tubular carcinoma (2), and secretory cancer (1). BPE = background parenchymal enhancement, IDC = invasive ductal carcinoma, DCIS = ductal carcinoma in situ, ILC = invasive lobular carcinoma, ER = estrogen receptor, PR = progesterone receptor, HER-2 = human epidermal growth factor receptor 2, TN = triple-negative, MRI = magnetic resonance imaging, NME = non-mass enhancement, and BCS = breast-conserving surgery.

**Table 2:** Concordance rates for assessment of the pathologic tumor extent using AB and FDP MRI according to the tumor subtypes.

			Tumor Extent Differences Between MRI and Pathology	Underestimation	Correct Estimation ^a^	Overestimation	*p ^b^*
			(mm)	N (%)	N (%)	N (%)	
Total (*n* = 330)	Reader 1	AB	3.3 ± 5.25	27 (8.2)	271 (82.1)	32 (9.7)	0.68
		FDP	5.2 ± 5.81	21 (6.4)	273 (82.7)	36 (10.9)	
	Reader 2	AB	3.8 ± 6.23	26 (7.9)	270 (81.8)	34 (10.3)	0.74
		FDP	4.5 ±6.14	22 (6.7)	272 (82.4)	36 (10.9)	
Histopathologic subtype							
IDC (*n* = 219)	Reader 1	AB	1.8 ± 9.8	12 (5.5)	193 (88.1)	14 (6.4)	0.72
		FDP	2.6 ± 8.5	9 (4.1)	195 (89.0)	15 (6.8)	
	Reader 2	AB	0.97 ± 10.2	11 (5.0)	194 (88.6)	14 (6.4)	0.85
		FDP	2.3 ± 12.6	10 (4.6)	194 (88.6)	15 (6.8)	
DCIS (*n* = 36)	Reader 1	AB	2.9 ± 13.5	5 (13.9)	21 (58.3)	10 (27.8)	0.59
		FDP	3.5 ± 12.8	4 (11.1)	20 (55.6)	12 (33.3)	
	Reader 2	AB	2.3 ± 12.6	4 (11.1)	20 (55.6)	12 (33.3)	0.77
		FDP	3.3 ± 14.5	3 (8.3)	21 (58.3)	12 (33.3)	
ILC (*n* = 45)	Reader 1	AB	−5.5 ± 11.2	8 (17.8)	33 (73.3)	4 (8.9)	0.78
		FDP	−3.8 ± 9.6	6 (13.3)	34 (75.6)	5 (11.1)	
	Reader 2	AB	−4.2 ± 12.1	7 (15.6)	34 (75.6)	4 (8.9)	0.84
		FDP	−2.5 ± 10.6	6 (13.3)	35 (77.8)	4 (8.9)	
Others (*n* = 30)	Reader 1	AB	1.1 ± 6.8	2 (6.7)	24 (80.0)	4 (13.3)	1
		FDP	1.4 ± 8.2	2 (6.7)	24 (80.0)	4 (13.3)	
	Reader 2	AB	0.5 ± 8.1	4(13.3)	22 (73.3)	4 (13.3)	0.89
		FDP	1.5 ± 8.3	3 (10.0)	22 (73.3)	5 (16.7)	
Molecular subtype							
Luminal A (*n* = 145)	Reader 1	AB	0.6 ± 8.7	11 (7.6)	122 (84.1)	12 (8.3)	0.83
		FDP	0.9 ± 11.5	9 (6.2)	122 (84.1)	14 (9.7)	
	Reader 2	AB	0.6 ± 9.8	10 (6.9)	121 (83.4)	14 (9.7)	0.88
		FDP	0.7 ± 10.6	9 (6.2)	122 (84.1)	14 (9.7)	
Luminal B (*n* = 124)	Reader 1	AB	0.6 ± 13.1	10 (8.1)	102 (82.3)	12 (9.7)	0.72
		FDP	0.5 ± 12.5	8 (6.5)	103 (83.1)	13 (10.5)	
	Reader 2	AB	0.7 ± 11.1	11 (8.9)	101 (81.5)	12 (9.7)	0.81
		FDP	0.3 ± 11.3	10 (8.1)	102 (82.3)	12 (9.7)	
HER-2 (*n* = 25)	Reader 1	AB	1.5 ± 13.1	6 (24.0)	12 (48.0)	7 (28.0)	0.73
		FDP	1.8 ± 14.5	4 (16.0)	13 (52.0)	8 (32.0)	
	Reader 2	AB	1.1 ± 12.9	5 (20.0)	12 (48.0)	8 (32.0)	0.76
		FDP	0.9 ± 16.1	3 (12.0)	13 (52.0)	9 (36.0)	
TN (*n* = 36)	Reader 1	AB	2.5 ± 7.7	0 (0.0)	35 (97.2)	1 (2.8)	NA
		FDP	3.3 ± 6.8	0 (0.0)	35 (97.2)	1 (2.8)	
	Reader 2	AB	1.7 ± 5.33	0 (0.0)	36 (100.0)	0 (0.0)	0.91
		FDP	3.4 ± 8.23	0 (0.0)	35 (97.2)	1 (2.8)	

Differences calculated as follows: tumor extents by MRI—tumor extent by pathology. ^a^ correct estimation is defined by a difference of ≤10 mm between the MRI-measured tumor extent and pathologically measured tumor extent. AB = abbreviated protocol, FDP = full diagnostic protocol, IDC = invasive ductal carcinoma, DCIS = ductal carcinoma in situ, ILC = invasive lobular carcinoma, HER-2 = human epidermal growth factor receptor 2, TN = triple-negative, and NA = not applicable. *^b^* the *p*-value is the result of a chi-square test.

**Table 3:** Agreement with the pathologic tumor extent using AB and FDP MRI according to the tumor subtypes.

			ICC *	*p*
Histopathologic subtype				
IDC (*n* = 219)	Reader 1	AB	0.68 [0.55, 0.75]	0.56
		FDP	0.74 [0.66, 0.80]	
	Reader 2	AB	0.67 [0.62, 0.73]	0.71
		FDP	0.70 [0.65, 0.75]	
DCIS (*n* = 36)	Reader 1	AB	0.80 [0.61, 0.89]	0.88
		FDP	0.81 [0.58, 0.91]	
	Reader 2	AB	0.77 [0.61, 0.88]	0.92
		FDP	0.78 [0.65, 0.90]	
ILC (*n* = 45)	Reader 1	AB	0.52 [0.25, 0.71]	0.45
		FDP	0.60 [0.40, 0.83]	
	Reader 2	AB	0.49 [0.21, 0.68]	0.52
		FDP	0.62 [0.33, 0.79]	
Others (*n* = 30)	Reader 1	AB	0.88 [0.70, 0.93]	0.56
		FDP	0.92 [0.83, 0.96]	
	Reader 2	AB	0.81 [0.72, 0.91]	0.81
		FDP	0.85 [0.76, 0.94]	
Molecular subtype				
Luminal A (*n* = 145)	Reader 1	AB	0.68 [0.58, 0.76]	0.51
		FDP	0.72 [0.66. 0.81]	
	Reader 2	AB	0.67 [0.52, 0.77]	0.75
		FDP	0.69 [0.63, 0.76]	
Luminal B (*n* = 124)	Reader 1	AB	0.68 [0.55, 0.75]	0.43
		FDP	0.73 [0.65, 0.82]	
	Reader 2	AB	0.67 [0.54, 0.73]	0.54
		FDP	0.71 [0.60, 0.81]	
HER-2 (*n* = 25)	Reader 1	AB	0.76 [0.43, 0.94]	0.88
		FDP	0.78 [0.54, 0.97]	
	Reader 2	AB	0.61 [0.27, 0.81]	0.51
		FDP	0.72 [0.42, 0.89]	
TN (*n* = 36)	Reader 1	AB	0.85 [0.68, 0.92]	0.78
		FDP	0.87 [0.71, 0.95]	
	Reader 2	AB	0.88 [0.67, 0.92]	0.72
		FDP	0.90 [0.76, 0.95]	

* Numbers in brackets are 95% confidence intervals of ICC values. AB = abbreviated protocol, FDP = full diagnostic protocol, ICC = intraclass correlation coefficient, IDC = invasive ductal carcinoma, DCIS = ductal carcinoma in situ, ILC = invasive lobular carcinoma, HER-2 = human epidermal growth factor receptor 2, and TN = triple-negative.

**Table 4:** Correlation coefficient with the pathologic tumor extent using AB and FDP MRI according to the tumor subtypes.

			Correlation Coefficient	*p*
Histopathologic subtype				
IDC	Reader 1	AB	0.69 [0.54, 0.77]	0.7
(*n* = 219)		FDP	0.74 [0.65, 0.78]	
	Reader 2	AB	0.7 [0.51, 0.73]	0.88
		FDP	0.72 [0.62, 0.87]	
DCIS	Reader 1	AB	0.7 [0.51, 0.72]	0.9
(*n* = 36)		FDP	0.71 [0.51, 0.79]	
	Reader 2	AB	0.6 [0.47, 0.73]	0.79
		FDP	0.62 [0.50, 0.77]	
ILC	Reader 1	AB	0.49 [0.44, 0.62]	0.47
(*n* = 45)		FDP	0.6 [0.46, 0.66]	
	Reader 2	AB	0.47 [0.45, 0.60]	0.49
		FDP	0.58 [0.51, 0.65]	
Others	Reader 1	AB	0.73 [0.71, 0.79]	0.56
(*n* = 30)		FDP	0.8 [0.77, 0.89]	
	Reader 2	AB	0.75 [0.73, 0.78]	0.83
		FDP	0.77 [0.75, 0.82]	
Molecular subtype				
Luminal A	Reader 1	AB	0.65 [0.62, 0.76]	0.88
(*n* = 145)		FDP	0.66 [0.63, 0.79]	
	Reader 2	AB	0.59 [0.55, 0.71]	0.63
		FDP	0.7 [0.66, 0.78]	
Luminal B	Reader 1	AB	0.72 [0.61, 0.80]	0.41
(*n* = 124)		FDP	0.69 [0.58, 0.79]	
	Reader 2	AB	0.7 [0.59, 0.82]	0.74
		FDP	0.72 [0.68, 0.81]	
HER-2	Reader 1	AB	0.66 [0.49, 0.70]	0.9
(*n* = 25)		FDP	0.68 [0.51, 0.72]	
	Reader 2	AB	0.53 [0.48, 0.67]	0.92
		FDP	0.55 [0.50, 0.68]	
TN	Reader 1	AB	0.79 [0.74, 0.83]	0.84
(*n* = 36)		FDP	0.81 [0.77, 0.89]	
	Reader 2	AB	0.81 [0.75, 0.85]	0.73
		FDP	0.84 [0.74, 0.88]	

AB = abbreviated protocol, FDP = full diagnostic protocol, IDC = invasive ductal carcinoma, DCIS = ductal carcinoma in situ, ILC = invasive lobular carcinoma, HER-2 = human epidermal growth factor receptor 2, and TN = triple-negative.

**Table 5:** Per-reader and per-protocol distribution of the CDR, PPV, and FDR for ipsilateral additional malignant lesions.

	Reader 1			Reader 2		
	AB	FDP	*p*	AB	FDP	*p*
CDR	66.66% (10/15)	86.66% (13/15)	0.71	60.00% (9/15)	66.66% (10/15)	0.89
PPV	90.90% (10/11)	81.25% (13/16)	0.9	50.00% (6/12)	58.82% (10/17)	0.77
FDR	9.10% (1/11)	18.75% (3/16)	0.52	50.00% (6/12)	41.17% (7/17)	0.12

AB = abbreviated protocol, FDP = full diagnostic protocol, CDR = cancer detection rate, PPV = positive predictive value, and FDR = false detection rate. The CDR was defined as the proportion of correctly detected additional malignant lesions relative to the total number of pathologically confirmed additional malignant lesions. The PPV was calculated as the proportion of correctly identified additional malignant lesions among all cases categorized as BI-RADS ≥ 4. The FDR represented the proportion of false positives out of the total cases categorized as BI-RADS ≥ 4. The *p*-values refer to the intrareader comparison of CDR, PPV, and FDR between AB- and FDP-MRI.

## Data Availability

Data available on request due to restrictions—The data presented in this study are available on request from the corresponding author due to patient's privacy, legal, or ethical reasons.

## Supplementary Materials

The following supporting information can be downloaded at https://www.mdpi.com/article/10.3390/diagnostics15212749/s1, Supplementary Materials and Methods [28,29,30], Table S1: Imaging sequences of full diagnostic protocol of breast MRI; Table S2: MRI morphology and pathologic results of additional ipsilateral malignant lesions.

## Abbreviations

AB-MRI	Abbreviated Magnetic Resonance Imaging
FDP-MRI	Full Diagnostic Protocol Magnetic Resonance Imaging
IDC	Invasive Ductal Carcinoma
DCIS	Ductal Carcinoma In Situ
ILC	Invasive Lobular Carcinoma
ER	Estrogen Receptor
PR	Progesterone Receptor
HER-2	Human Epidermal Growth Factor Receptor 2
TN	Triple-Negative
ICC	Intraclass Correlation Coefficient
CDR	Cancer Detection Rate
PPV	Positive Predictive Value
FDR	False Detection Rate
MIP	Maximum Intensity Projection
BI-RADS	Breast Imaging Reporting and Data System
