# Supplementary material for: Comparison of Abbreviated MRI and Full Diagnostic Protocol MRI for Surgical Planning in Patients with Newly Diagnosed Breast Cancer
Source: Diagnostics (Basel). 2025 Oct 30;15(21):2749. doi: 10.3390/diagnostics15212749 (PMC12609794; doi:10.3390/diagnostics15212749)
Supplement: Supplementary file 1 [file diagnostics-15-02749-s001.zip › diagnostics-3904991-supplementary.pdf]

**Supplementary Materials and Methods.**

The Allred score was utilized to quantify ER and PR expressions, with any total Allred score >2 classified as ER- or PR-positive [28]. A HER-2 score of 0 or 1 was labeled as negative, while a score of 3 was deemed positive. An equivocal HER-2 score of 2 prompted the performance of silver-enhanced *in situ* hybridization, and the HER-2/chromosome enumeration probe 17 (CEP17) ratio of ≥2.0 or the HER-2/CEP17 ratio of <2.0 accompanied by an average HER-2 copy number ≥6.0 was deemed positive [29]. A Ki-67 cut-off value of 14% is used to differentiate luminal A from luminal B, with values <14% indicating luminal A and ≥14% indicating luminal B [30].

**Supplementary Table 1.** Imaging sequences of full diagnostic protocol of breast MRI

| | T1-weighted imaging | T2-weighted imaging | DWI[a] | DCE[b] |
|---|---|---|---|---|
| Plane | axial | axial | axial | axial |
| TR/TE (msec) | 746/10 | 8087/88 | 550/50 | 4/2 |
| Matrix (pixels x pixels) | 352 × 256 | 384 × 256 | 100 × 128 | 288 × 416 |
| Slice thickness (mm) | 3 | 3 | 3 | 1 |

TR repetition time, TE echo time, DWI diffusion-weighted image, DCE dynamic contrast-enhanced image.

[a]b-values of DWI were 0 and 800 sec/mm$^2$.

[b]DCE included one precontrast phase and four or five postcontrast phases using 3D-gradient echo and fat-suppressed with flip angle of 15°.

**Supplementary Table 2.** MRI morphology and pathologic results of additional ipsilateral malignant lesions

| Case No. | MRI morphology | Size (mm) | Pathology |
|---|---|---|---|
| 1 | Mass | 7 | IDC |
| 2 | NME | 9 | ILC |
| 3 | Mass | 8 | IDC |

| | | | |
|---|---|---|---|
| 4 | Mass + NME | 20 | ILC |
| 5 | Mass | 6 | DCIS |
| 6 | NME | 15 | IDC |
| 7 | Mass | 7 | IDC |
| 8 | Mass | 9 | IDC |
| 9 | Mass | 11 | IDC |
| 10* | Mass | 5 | IDC |
| 11* | NME | 17 | DCIS |
| 12 | Mass | 10 | ILC |
| 13 | Mass | 6 | IDC |
| 14 | Mass | 9 | IDC |
| 15 | Mass | 5 | IDC |

MRI = magnetic resonance imaging; IDC = invasive ductal carcinoma; ILC = invasive lobular carcinoma; DCIS = ductal carcinoma in situ; NME = non-mass enhancement. * = Two of the additional malignant lesions were not detected on AB-MRI, although those were found on FDP-MRI.
